# Prediction of mining-induced subsidence at Barapukuria longwall coal mine, Bangladesh

**DOI:** 10.1038/s41598-022-19160-1

**Published:** 2022-08-30

**Authors:** A. K. M. Badrul Alam, Yoshiaki Fujii, Shaolin Jahan Eidee, Sophea Boeut, Afikah Binti Rahim

**Affiliations:** 1 Department of Petroleum and Mining Engineering, Faculty of Civil Engineering, MIST, 1st Floor, General Mustafiz Tower, Mirpur Cantonment, Dhaka, 1216 Bangladesh; 2 Faculty of Engineering, Hokkaido University, Sapporo, 060-8628 Japan; 3 Institute of Technology of Cambodia, P.O. Box 86, Phnom Penh, 12156 Cambodia; 4 School of Civil Engineering, Universiti Teknologi Malaysia, 81310 Johor Bahru Johor, Malaysia

**Keywords:** Civil engineering, Environmental impact

## Abstract

It is essential to predict the mining-induced subsidence for sustainable mine management. The maximum observed subsidence having a noticeable areal extent due to Northern Upper Panels (NUP) and Southern Lower Panels (SLP) at the Barapukuria longwall coal mine is 5.8 m and 4.2 m, respectively, after the extraction of a 10 m thick coal seam. The mining-induced subsidence was simulated by the Displacement Discontinuity Method. The numerical model considered the effects of the ground surface, mining panels, faults, and the dyke. The predicted and the observed subsidence due to the mining of NUP and SLP were compared by varying Young's modulus, and the 0.10 GPa Young's modulus was found to be the best match in the geo-environmental condition. The effects of the faults and the dyke in the calculation were negligible. Future subsidence was predicted by considering 30 m extraction of the thick coal seam as 15.7–17.5 m in NUP and 8.7–10.5 m in SLP. The vulnerable areas demarcated considering the tilt angle and extensile strain might extend up to the coal mine office area and some villages.

## Introduction

Subsidence is allowed in the longwall coal mining method; the stress-induced accidents are lower in this mining system, with a higher production rate. As subsidence is a must in a longwall coal mine without stowing, it is essential to predict the mining-induced subsidence for sustainable mine management. Material extraction by longwall mining may induce several types of ground movements, such as vertical ground displacements, ground curvature (tilt angle), and horizontal ground strain (extensile strain) at the surface^1^. Buildings and infrastructures on the surface might be damaged^2^ depending upon the position. Surface subsidence was first observed in 2006, evident from cracks in the surface structures of the Barapukuria (Bangladesh) mining area, and the government has acquired 2.61 km^2^ of the affected land area^3,4^.

**Figure 1:**
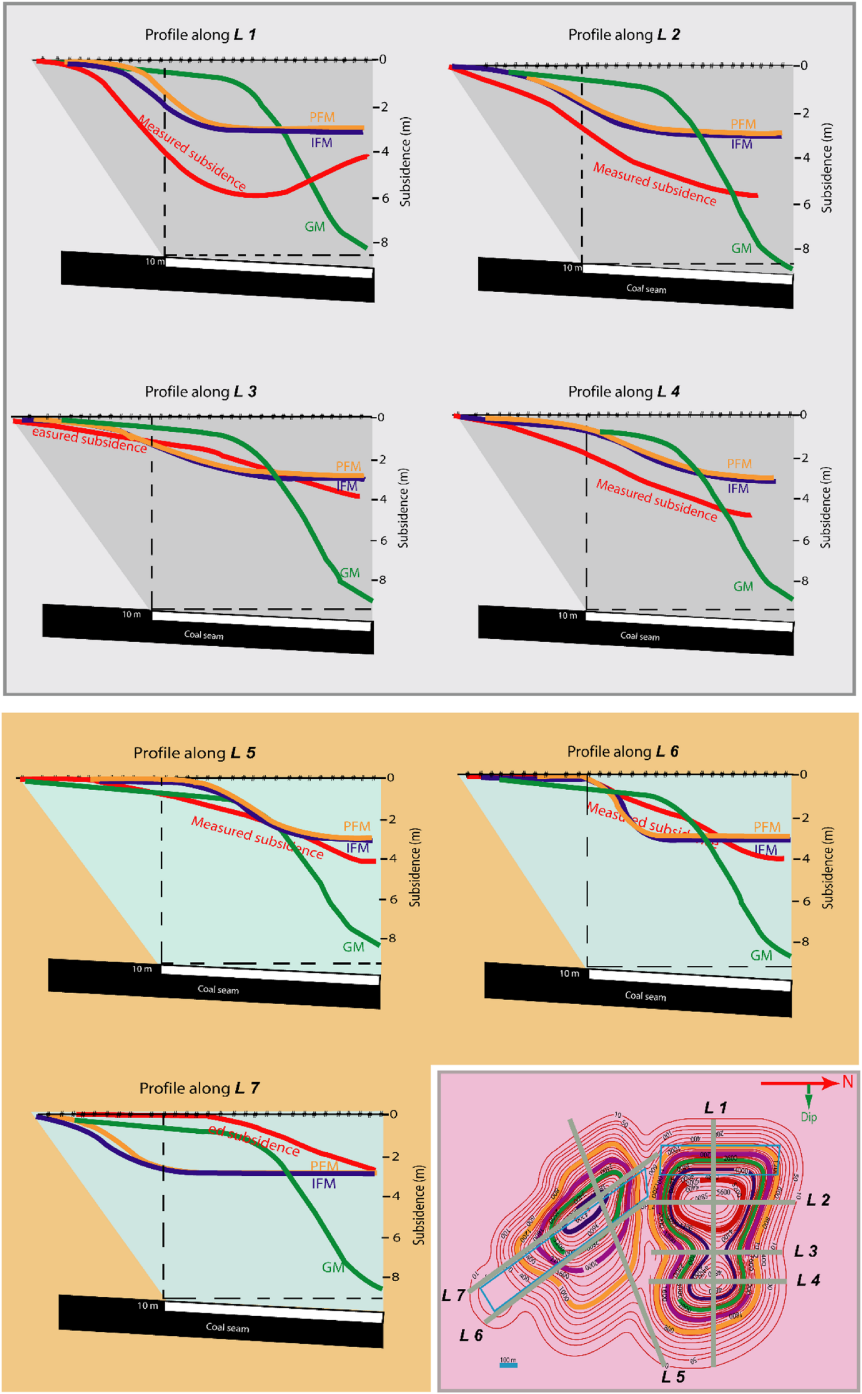
Predicted and measured subsidence profiles by empirical methods. GR_Graphical Method; PFM_Profile Function Method; IFM_Influence Function Method. (Modified after Ahmed^9^).

Several prediction methods have been developed and classified into empirical, semiempirical, and numerical methods and are the graphical methods, profile function methods, and influence function methods. Graphical methods (GM) are derived from extensive field data by the NCB^5^ concerning a particular geo-environment context. The profile function method (PEM) follows a curve-fitting procedure to match the predicted profile with observed profiles by mathematical functions^6^. These methods suffer from the same disadvantage as the graphical methods having many profile functions^5^: they can be used for a particular geo-environmental condition and are developed to predict a two-dimensional subsidence profile. The superposition principle is used in the Influence function methods (IFM) developed by Ren, Reddish, and Whittaker^7^ and extensively used^8^ to predict mining subsidence. Different coefficients are suggested to adjust the superposition with exact questionable influence and application fields^8^. The subsidence was tried to predict by the empirical methods (Fig. 1) due to longwall coal mining at Barapukuria, Bangladesh^9^. It was found that the shape and magnitude are different from the measured subsidence in GM method; PFM and IFM predicted subsidence profile shape is near to measured subsidence with different magnitude for NUP for all the lines; PFM and IFM predicted subsidence profile is near the measured subsidence with different in shape for 5 and 6 lines but different for line 7 for SLP. The observed and the predicted subsidence showed a noticeable miss-match and might not be suitable for the geo-environmental condition.

Numerical methods like the finite element method (FEM)^10^, the distinct element method or the finite differences method^11,12^ physical models^13,14^ and, GIS and remote sensing^15^ have been used for subsidence prediction in the different mines and can be very accurate when validated. However, a mine-wide 3-D FEM analysis would cost much and be time-consuming. Introducing a mined-out area closure would be difficult, and faults and dykes may need special elements. In this research, we have tried to predict the mining-induced subsidence of the Barapukuria longwall coal mine by the Displacement Discontinuity Method (DDM) because it can easily handle the effect of the ground surface, closure of mined-out areas, and deformation of faults and dykes. DDM was originally developed by Crouch and Fairhurst^16^ as a boundary element method (BEM), especially for applying to tabular excavations. They presented algorithms to effectively obtain elastic solutions for mine-wide stress change due to the mining of parallel ore seams. In the algorithm, parallel ore seams are divided into square displacement discontinuity (DD) elements, and boundary conditions are assigned according to the mining indices, unmined, mined, or closed. Simultaneous equations, each representing stress change in an infinite elastic body by a DD element, are solved to obtain the elastic solution.

The current authors modified the above method so that the ground surface, mining panels, faults, and dykes at any orientations could be divided by rectangular DD elements and used here. The predicted and the observed subsidence were compared, and the future subsidence and the approximate vulnerable areas are demarcated for the geo-environmental condition.

## Structural framework and the characteristics

**Figure 2:**
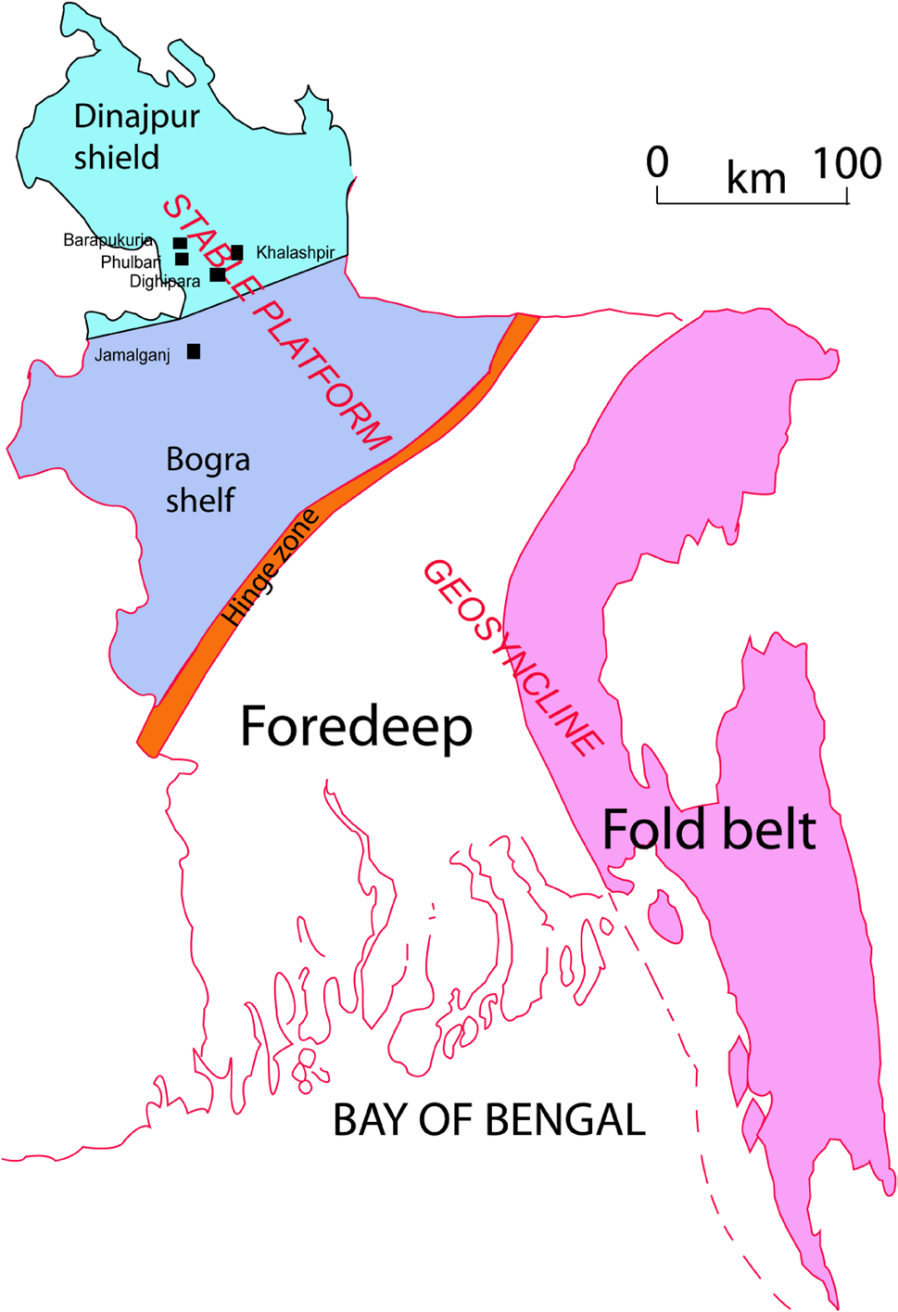
Structural framework and the coal basins of Bangladesh. (Modified after Imam^17^). (Abobe Illustrator 10, https://www.adobe.com/products/illustrator.html).

Tectonically Bangladesh can be broadly subdivided into two zones (i) Stable Platform (SP) (ii) Geosynclinal Basin (GB) that are separated by a narrow northeast-southwest trending shelf edge/ slope break known as Hinge Zone (HZ)^17^ (Fig. 2). The SP is relatively geologically stable and is situated in the northern part of the HZ. The GB is in the south, characterized by thick sedimentary rock layers resulting from rapid subsidence and sedimentation in a relatively short span of geological time.

The SP and GB can be sub-divided into two subzones each; Dinajpur shield and Bengal shelf in SP; Folded belt and Foredeep in GB. The Dinajpur shield^18^ has a thin sedimentary cover above the Precambrian basement rock, whereas the Bogra shelf has moderately thick sedimentary rock layers gently dipping towards the HZ. Folds characterize the Folded belt, and the intensity of the folding is greater in the eastern part compared to the western part of thick sedimentary rock layers. The Foredeep zone is characterized by horizontal to sub-horizontal relatively thick sedimentary rock layers without major tectonic deformation.

Five coal basins, namely Barapukuria, Phulbari, Khalashpir, Dighipara, and Jamalgonj, have been discovered in the SP of Bangladesh^17^. Among them, the Barapukuria coal basin, where the only coal mine is being operated, is situated in the Dinajpur shield, where the coal seams are in relatively shallow depths starting from 131 to 328 m depending upon the coal basins. The Jamalgonj coal basin is in the Bogra shelf, where the coal seam is encountered at a relatively deeper depth of 640 m.

Barapukuria coal basin is a graben, an asymmetrical faulted syncline (Fig. 2), with an approximately N-S axis^19^. The rock sequence of the coal basin consists of the following five units^19^. (1) Madhupur Clay Formation (MC) (2) Upper Dupi Tila Formation (UDT) (3) Lower Dupi Tila Formation (LDT) (4) Gondwana Formation (GW), and (5) Basement Complex (BC).

**Figure 3:**
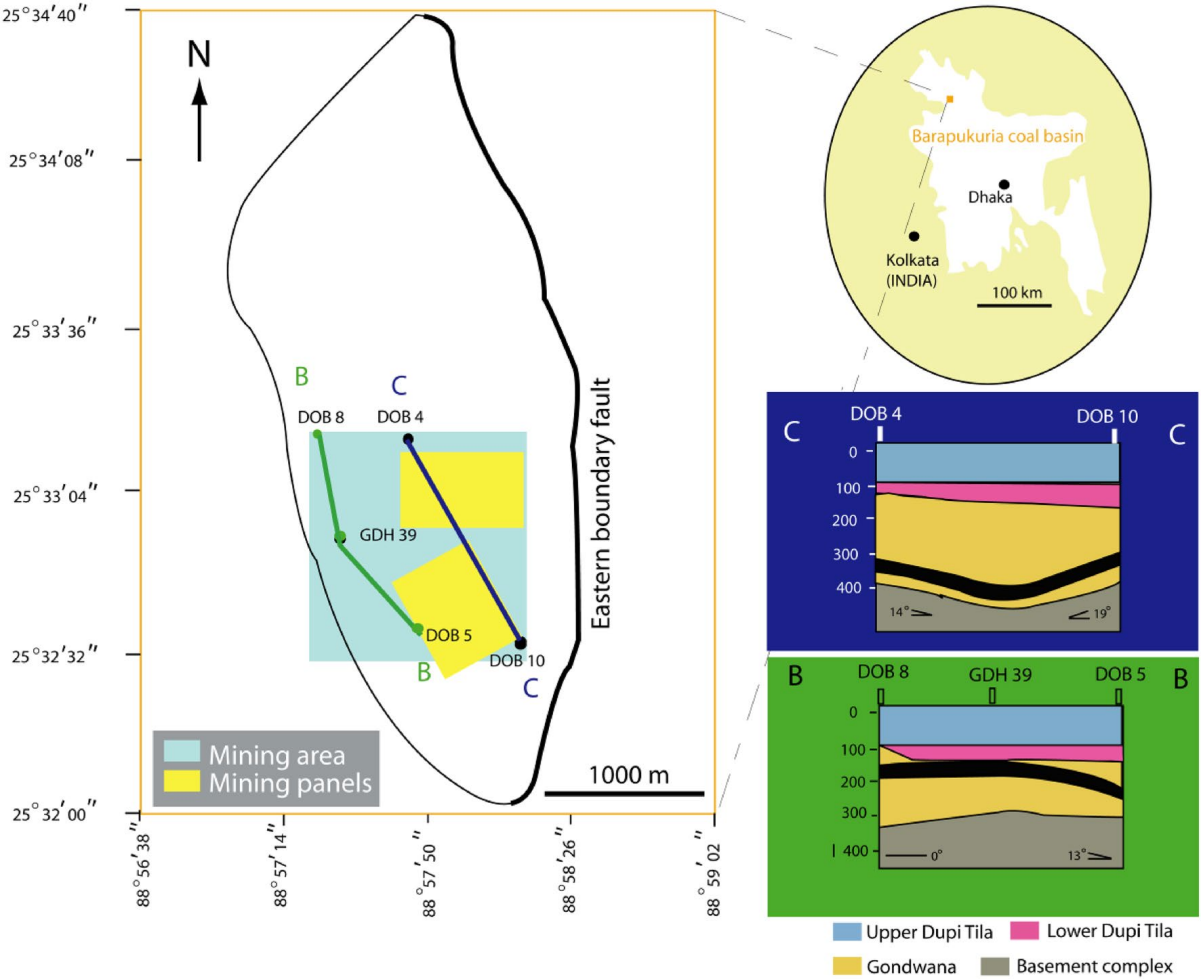
(**a**) Barapukuria coal basin and the areal extent of mining and (**b**) the associated rock layers with the thickest coal seam. (Modified after Armstrong^19^). (Rockworks20, https://www.rockware.com/product/rockworks/#; Abobe Illustrator 10, https://www.adobe.com/products/illustrator.html).

The MC is Holocene to recent in age and about 1–15 m thick^20^. The MC is underlain by DT, mainly a Late Miocene –Middle Pliocene aged layer. The UDT is mainly an unconsolidated to partly consolidated sand layer; with medium to coarse-grained, occasionally gravelly with bands of silt with an average thickness of about 94–126 m in the basin^19,20^, having a thickness of almost 100 m in the mine area (Fig. 3). The LDT consists of sandstone, silt, and white clay. The thickness varied from 0 to 80 m in the basin^19,20^, which is 0 to 60 m in the mine area (Fig. 3). The DT is underlain by GW, a Permian-aged coal-bearing rock layer unconformable on the Basement Complex. This rock sequence is up to 390 m thick^19,20^ in the basin, about 150–300 m in the mine area (Fig. 3), consisting of predominantly arkosic sandstone with subordinate siltstones, shales, and breccia-conglomerates with occasional interbedded siltstone, sandstones^20^. The coal seams are found in the GW. The average thickness of the thickest coal seam of the basin is about 36 m. The coal seam has a gentle slope of 13–19°, dipping towards the east. The BC is mainly a layer of diorite, meta-diorite, ophlitic gneiss, and granite rock^20^.

**Figure 4:**
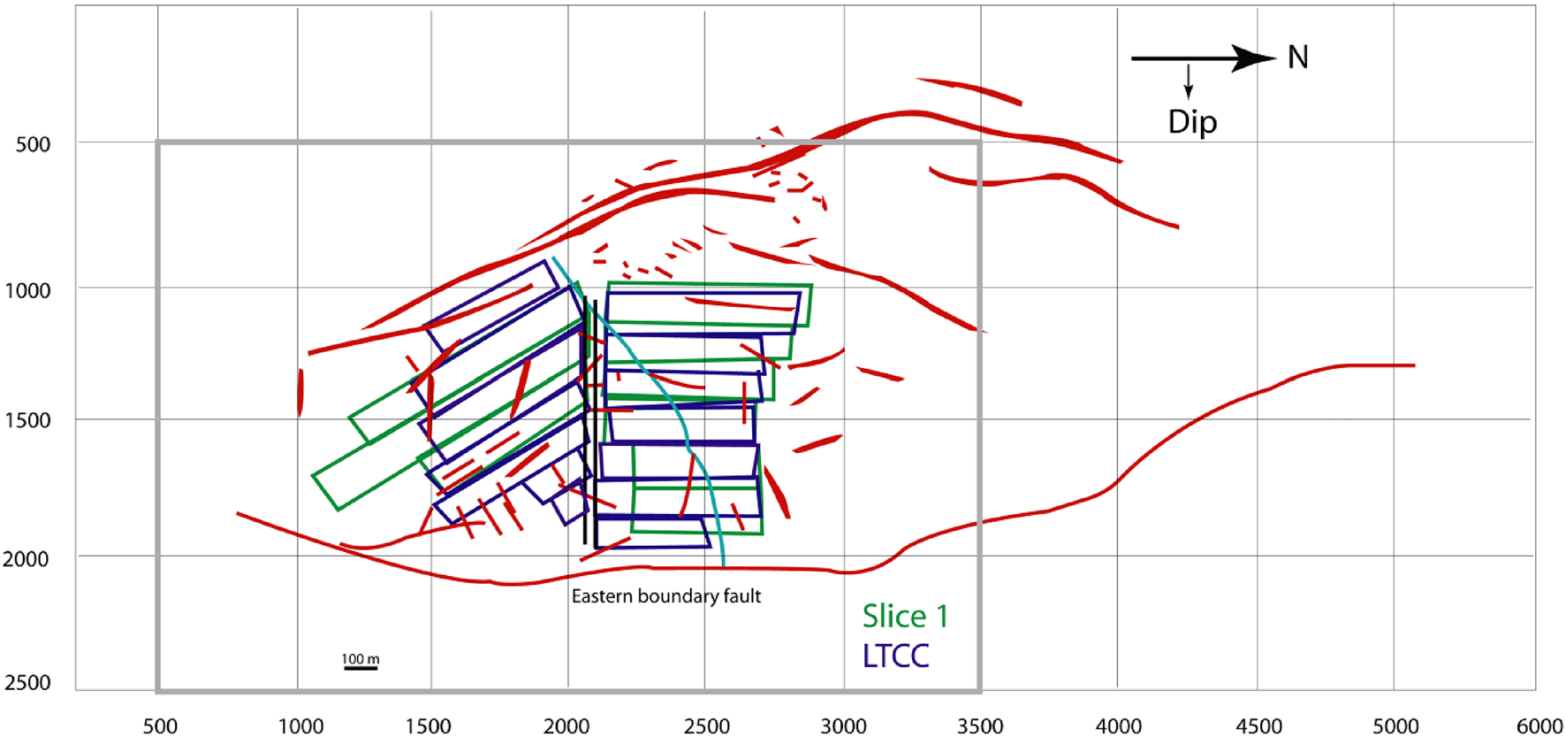
The faults (red) and a dyke (cyan) of the Barapukuria coal basin and the mining panels (BCMCL^21^).

The western part is more faulted than the southern part of the Barapukuria coal basin^21^ (Fig. 4). Faults bound the basin east by Eastern Boundary Fault (EBF) and west by numerous. The faults within the basin can be divided into (i) intra-basinal faults and (ii) boundary faults. The EBF is downthrown at 70–75° in the west and has a vertical displacement of about 200 m is around 5 km in length with NNW-SSE and N-S strike^21^. The faults of the west have the strike mainly of NNW-SSE and some portion of about NNE-SSW. There are several intra-basinal faults with the throw about 10 m within the coal-bearing rock layer in the mine area. A dyke, an igneous intrusion, has been detected in the northern mining panels with a strike of around NEE-SWW.

**Figure 5:**
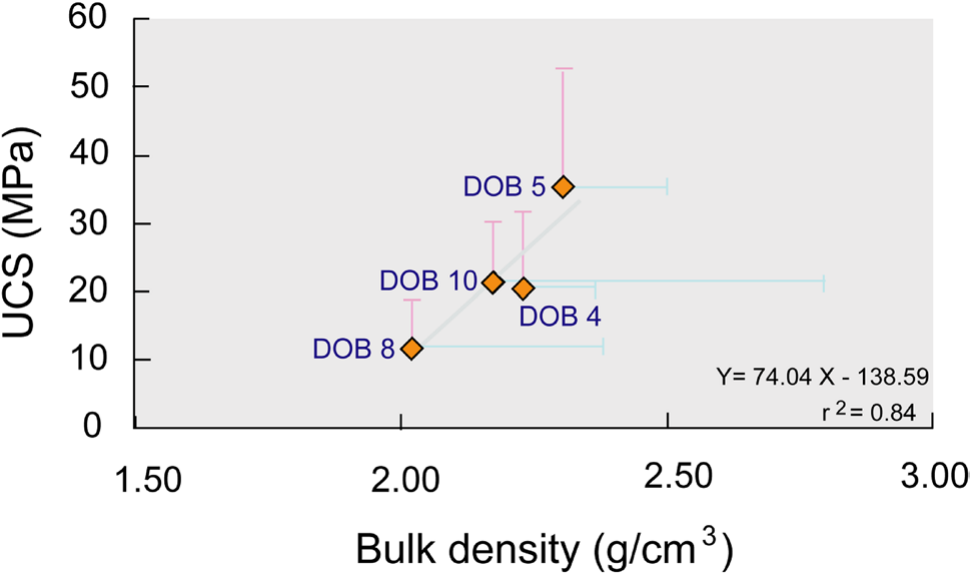
(**a**) Uniaxial compressive strength and (**b**) bulk density of the coal bearing formation.

The uniaxial compressive strength (UCS) of the coal-bearing rock (GW) (Fig. 5) is relatively high, 35.61 ± 17.08 MPa (*n* = *3*), with a bulk density of 2.30 ± 0.20 g/cm^3^ (*n* = *3*) in DOB 5, which is the southern up-dip portion of the basin. The UCS is moderately ranged from 20.91 ± 11.22 MPa (*n* = *10*) with bulk density 2.22 ± 0.15 g/cm^3^ (*n* = *10*) in DOB 4 to 21.57 ± 8.98 MPa (*n* = *7*) with bulk density 2.17 ± 0.62 g/cm^3^ (*n* = *7*) in DOB 10 which represents the almost central and southern down-dip portion of the basin. In the central up-dip portion represented by DOB 8, the UCS is the lowest of 12.34 ± 6.61 MPa (*n* = *6*) with a bulk density of 2.02 ± 0.36 g/cm^3^ (*n* = *6*)^19^.

Many faults (Fig. 4) with weaker rocks (Fig. 5) are in the central up-dip portion than those of the central and southern portion of the basin, where few faults with stronger rocks are. The ground in the south is relatively stronger than that of the central part of the basin. The central up-dip ground is weaker than that of the central portion of the basin.

## Materials and methods

### The observed subsidence

**Figure 6:**
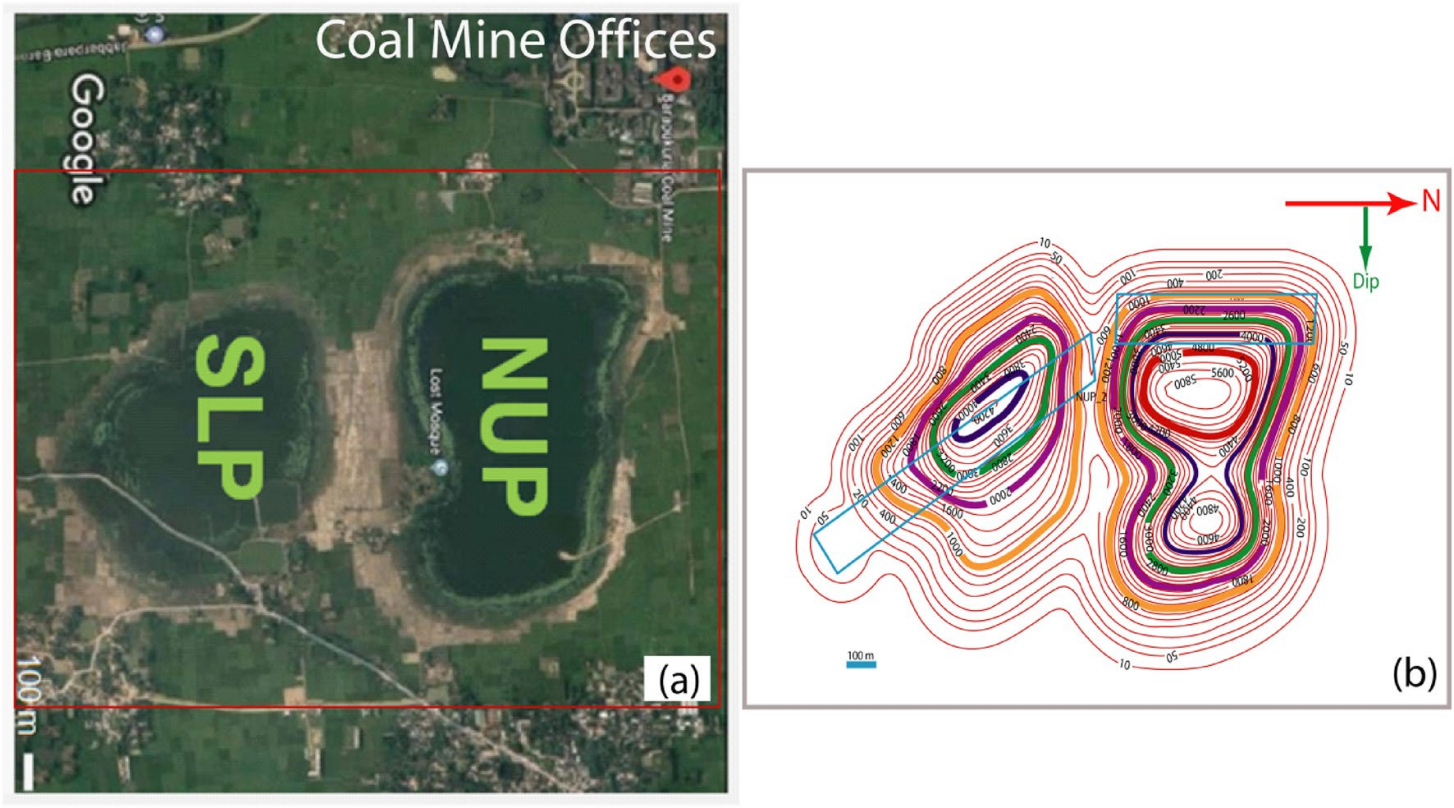
The subsidence in the Barapukuria coal mine area. (**a**) Subsidence epicenters (Google^22^ image) (**b**) Subsidence (mm) contour (BCMCL^21^). (Abobe Illustrator 10, https://www.adobe.com/products/illustrator.html).

From the bird’s eye view of the Barapukuria coal mine area, the subsided area can be divided into two regions (Fig. 6a), i.e., the northern and southern parts considering the subsidence epicenters^22^. The subsidence in the north is just above the Northern Upper Panels and is named NUP, and it is above the Southern Lower Panels in the south, named SLP. The observed subsidence is shown as a contour map in Fig. 6b^23^. The subsidence in the north can be further subdivided into North-Western and North-Eastern zones. The maximum subsidence in the North-Western and the North-Eastern zones is 5.8 m and 4.6 m, respectively (Fig. 6b), whereas; it is 4.2 m in the southern part. The observed subsidence of the contour map was converted to grid values having a specific range in the modeled grid area to compare the observed and the predicted subsidence.

### Modification of the DDM method and assigned boundary conditions

The algorithm by Crouch and Fairhurst^16^ focuses on effectively obtaining mine-wide stress distribution by mining parallel tabular ore seams with limited computer resources at the time of publication. We modified the algorithm so that non-parallel rectangular elements could be used.

Boundary conditions are as follows. *x*- and *y*-axes are in the strike and dip directions. *z*-axis is normal to the seam, fault, or dyke. *b* is the displacement discontinuity. *b*_x_ and *b*_y_ represent slip along *x*- and *y*-axes. Positive or negative *b*_z_ represents opening or closure of the roof and floor, fault surfaces, or dyke surfaces. For the ground surface and the mined coal seam elements,

1 $$ \tau_{zx} = \tau_{xy} = \sigma_{z} = 0 $$

where *τ* and *σ* are shear and normal stress. If − *b*_3_ exceeds the adjusted working height of the coal seam elements,

2 $$ b^{\prime}_{z} = - \alpha t $$

where *α* and *t* are a coefficient and the working height. And

3 $$ \begin{aligned} b^{\prime}_{x} = & b_{x} \frac{{b^{\prime}_{z} }}{{b_{z} }} \\ b^{\prime}_{y} = & b_{y} \frac{{b^{\prime}_{z} }}{{b_{3} }} \\ \end{aligned} $$

However,

4 $$ \begin{aligned} {\text{If }}\tau_{\max } = & \sqrt {\tau_{zx}^{2} + \tau_{yz}^{2} } \ge - \sigma_{z} \tan \phi , \\ \tau^{\prime}_{zx} = & \tau_{zx} \frac{{ - \sigma_{z} \tan \phi }}{{\sqrt {\tau_{zx}^{2} + \tau_{yz}^{2} } }} \\ \tau^{\prime}_{xy} = & \tau_{yz} \frac{{ - \sigma_{z} \tan \phi }}{{\sqrt {\tau_{zx}^{2} + \tau_{yz}^{2} } }} \\ \end{aligned} $$

where *ϕ* is the friction angle. For unmined elements,

5 $$ \begin{aligned} \tau_{xz} = & \frac{{b_{x} }}{t}G \\ \tau_{yz} = & \frac{{b_{y} }}{t}G \\ G = & \frac{E}{2(1 + \nu )} \\ \sigma_{z} = & \frac{{b_{z} }}{t}E \\ \end{aligned} $$

where *G*, *E*, and *ν* are the shear modulus, Young's modulus, and Poisson's ratio, respectively. For faults and dykes Eq. (4) is used.

### The model and the simulation

**Figure 7:**
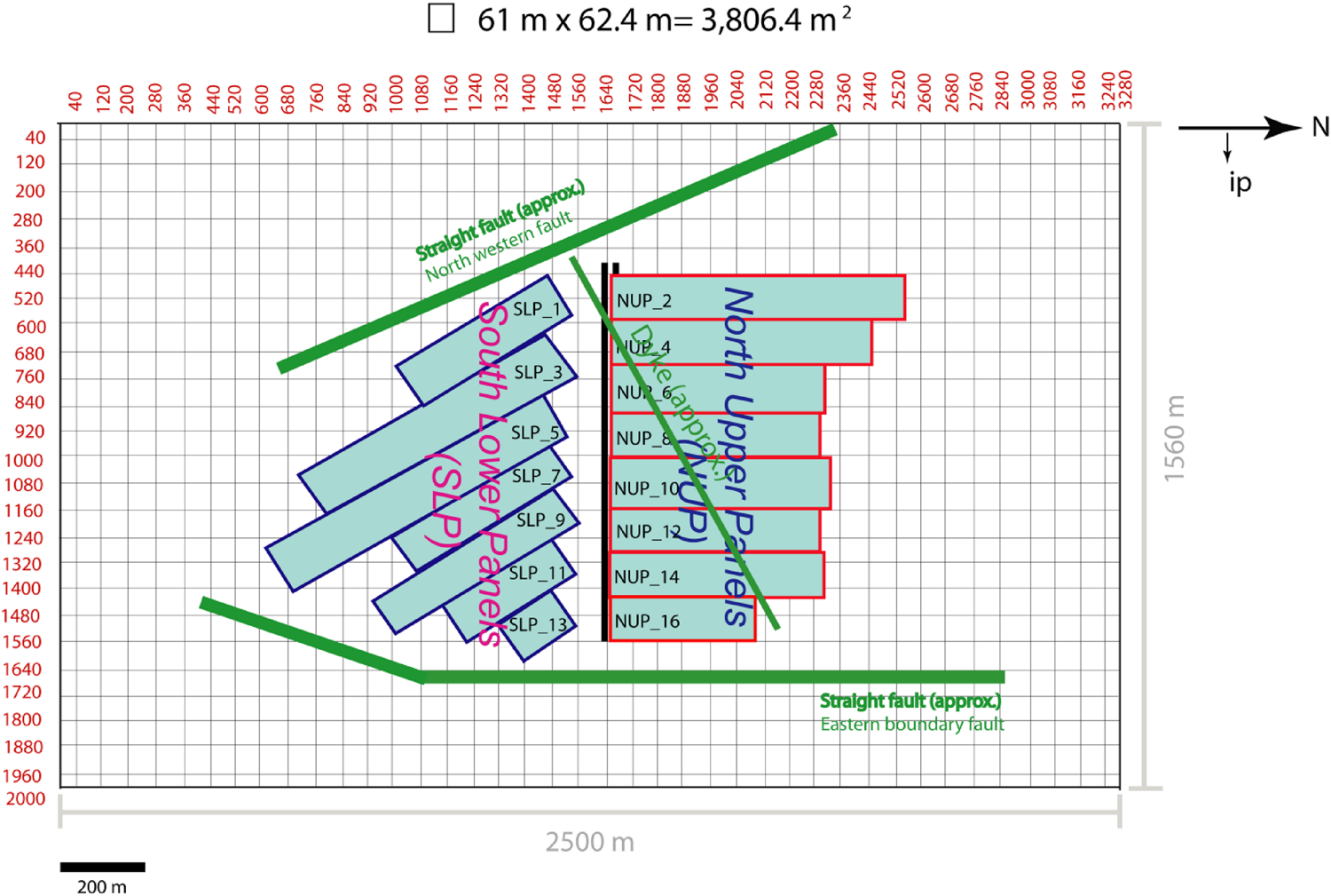
The mine model with mine panels and main discontinuities.

The ground surface of 2500 × 1560 m^2^ was divided by 40 × 25 DD elements, and the free surface condition was assigned. Each mining panel, fault, or dike was approximated by a rectangular plane (Fig. 7) divided into 4–22 DD elements. The element division is not fine enough due to the memory limitation. One of the reasons is that the matrix to be solved BEMs, including DDM, is not sparse, and techniques such as the band matrix method for FEM cannot be used. Also, the used compiler (Microsoft FORTRAN Power Station, ver.4.0) is not the latest version and generates only 32-bit executables. This problem should be solved in the future.

The mining height was assigned as 10 m on average, the first 3 m slice of coal was extracted by conventional longwall mining, and the next 7 m slice was extracted by the longwall top coal caving (LTCC) method. A friction angle of 30° was assigned to the faults and the dyke.

**Figure 8:**
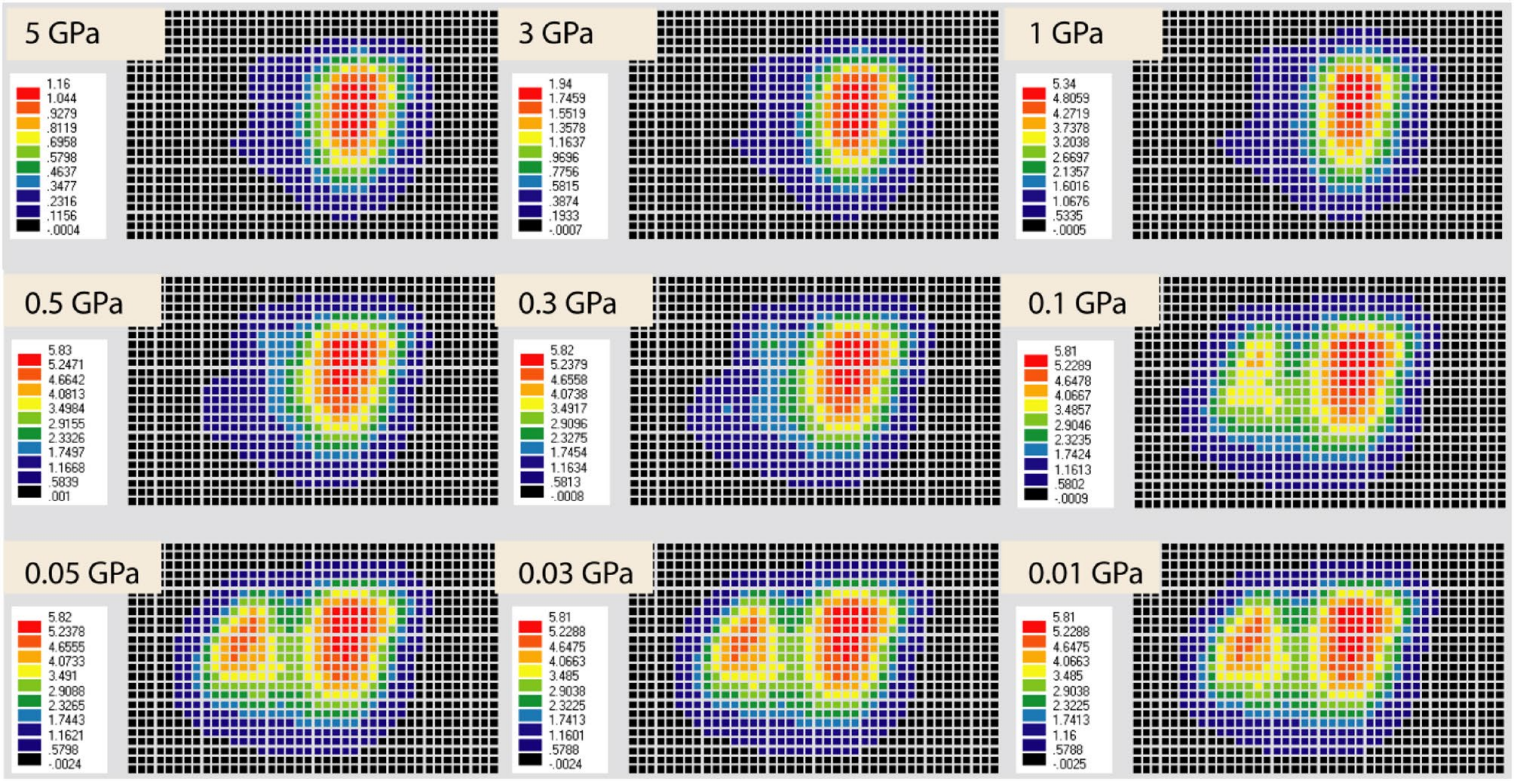
Calculated subsidence (m) considering Young’s modulus of (**a**) 5 GPa (**b**) 3 GPa (**c**) 1 GPa with a logarithmic decrement. (ev ver5.01 http://fubuki.g1.xrea.com/rml/fujii/ev/ev.htm).

The calculation should be carried out for the case in which the ground surface, mining panels, faults, and the dyke existed (Case1) and the case without mining panels (Case2), and subsidence for Case2 was subtracted from that for Case1 to obtain subsidence by mining panels. However, calculation with the ground surface and mining panels (Case3) was carried out first for simplicity. The calculated results show a peak at NUP and another peak at SLP for lower Young's modulus, and only one peak at NUP for higher Young's modulus (Fig. 8).

## Results and discussions

### The selection of the best value for young’s modulus

**Figure 9:**
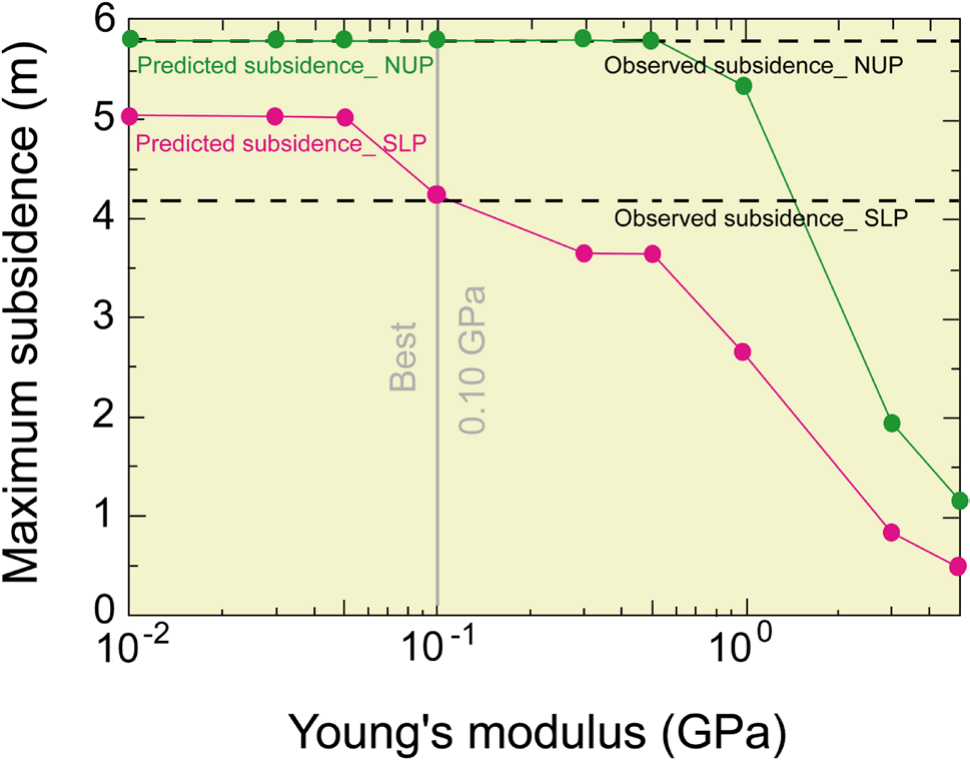
Young’s modulus effect in the predicted subsidence.

The Young's modulus of rock, rock-like material, and rock mass varies with environmental conditions^24–34^. It is also known that Young's modulus of the rock mass is much smaller than Young's modulus of intact rock specimens. In other words, it is not easy to deterministically fix Young's modulus value. The selection of Young's modulus was performed by back analysis. The peak values are saturated by the closure of the mining panels for lower Young's modulus and decrease with Young's modulus (Fig. 9). As a result of comparing the calculated results with the observation, Young's modulus of 0.1 GPa was selected as the best value. The predicted subsidence distribution (Fig. 8, 0.1 GPa) well simulated the observed one (Fig. 6) with a slightly different areal extent.

### Effects of the faults and the dyke

**Figure 10:**
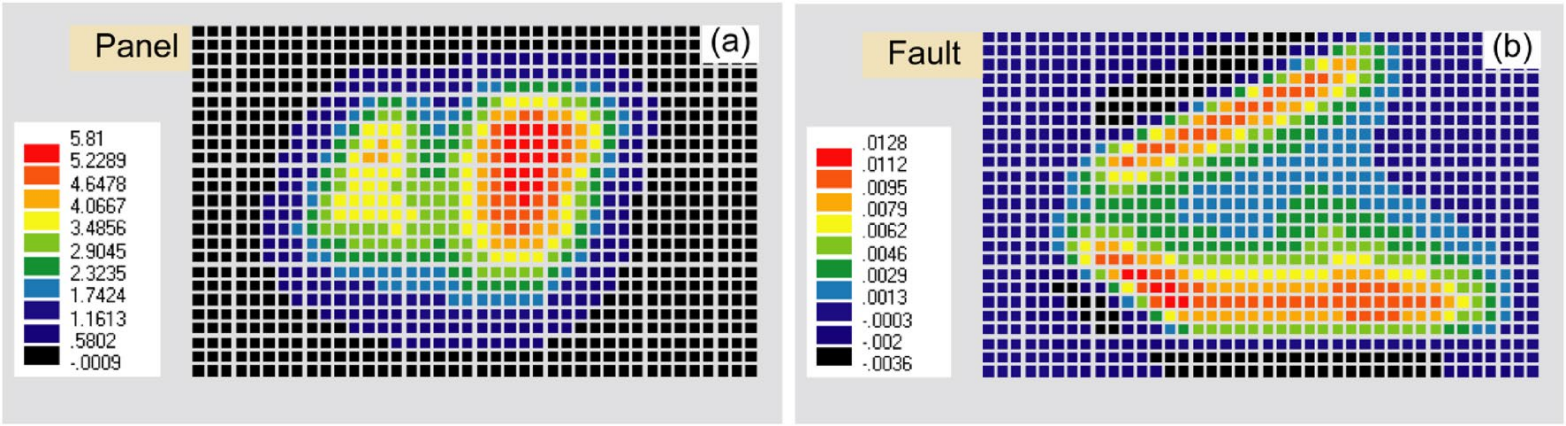
(**a**) The subsidence (m) due to panel extraction with faults and the dyke effect and (**b**) faults and the dyke affect. (ev ver5.01 http://fubuki.g1.xrea.com/rml/fujii/ev/ev.htm).

The subsidence due to mining panels, faults, and the dyke (Fig. 10a) is almost the same as the subsidence without faults and the dyke (Fig. 8, 0.1 GPa). The contribution by the faults and the dyke is almost negligible (Fig. 10b).

### Future subsidence and vulnerable area

**Figure 11:**
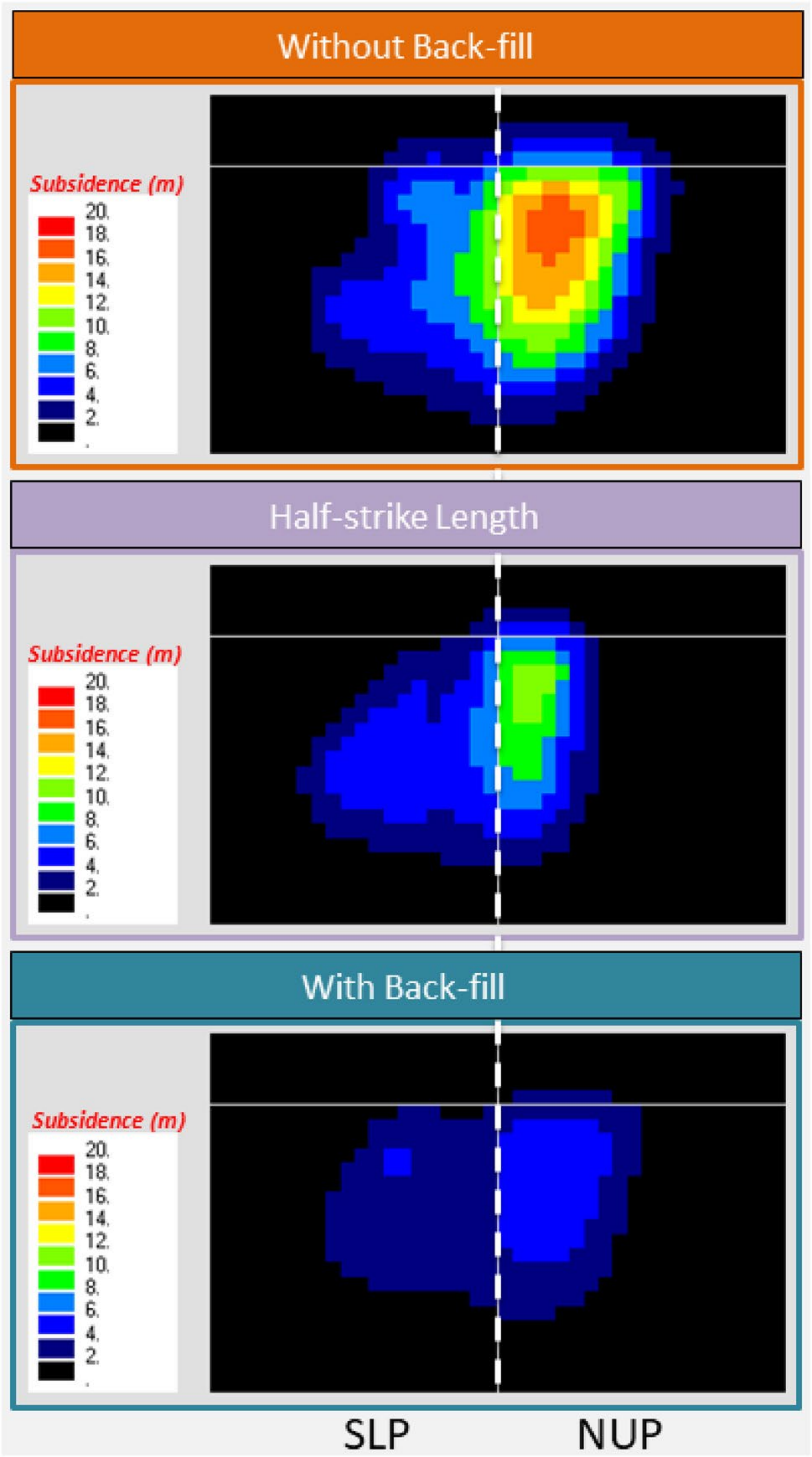
Future subsidence (m) due to total extraction of 30 m coal seam. (ev ver5.01. http://fubuki.g1.xrea.com/rml/fujii/ev/ev.htm).

**Figure 12:**
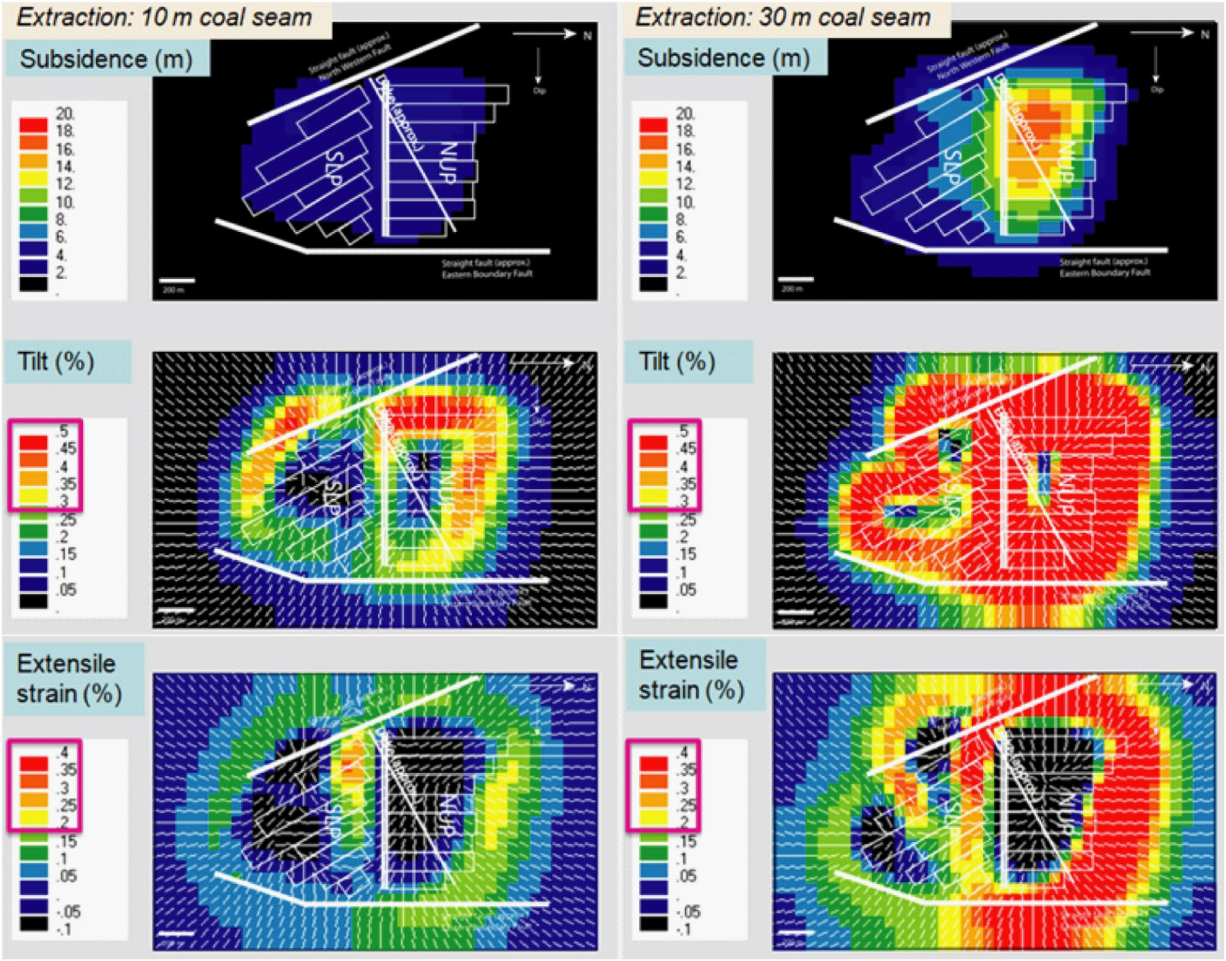
Future subsidence and vulnerable area considering tilt angle and extensile strain due to total extraction of 30 m coal seam. (ev ver5.01. http://fubuki.g1.xrea.com/rml/fujii/ev/ev.htm).

**Figure 13:**
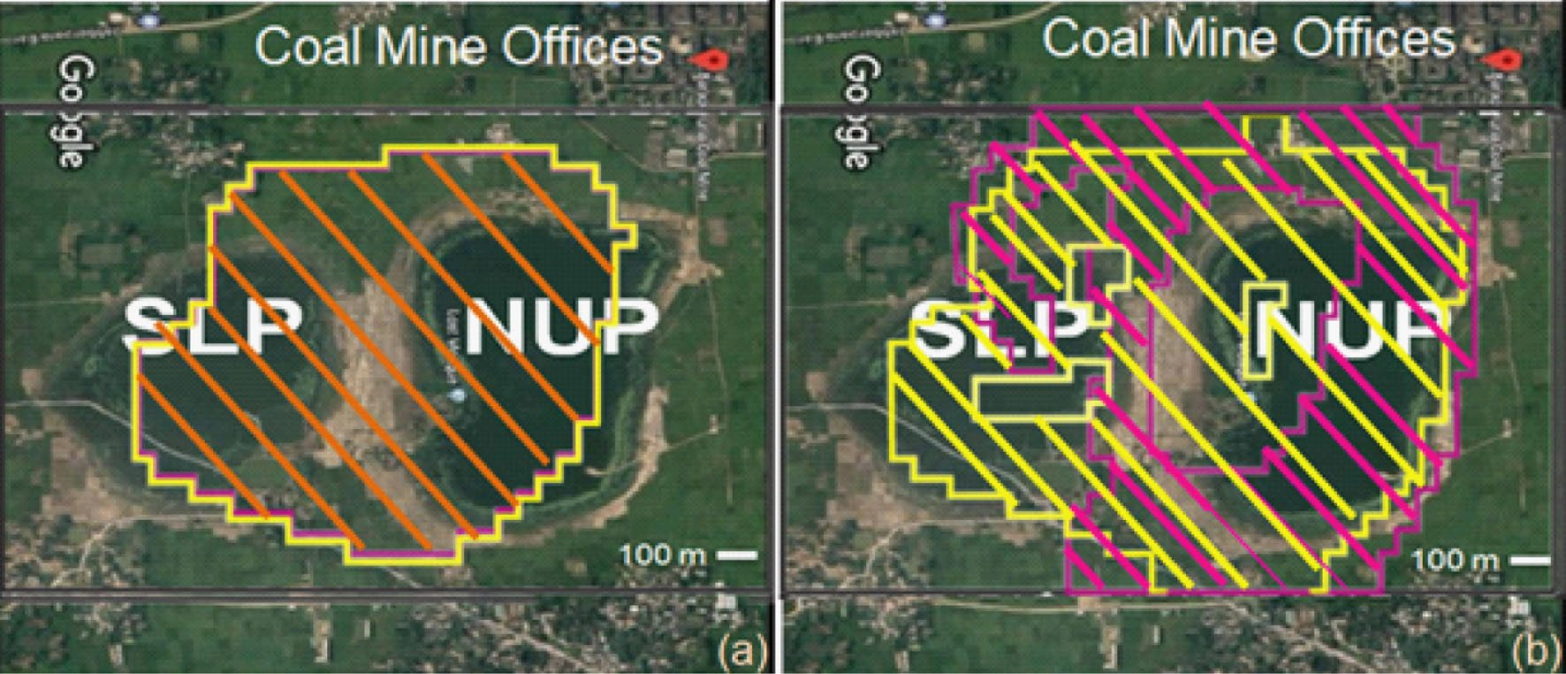
Future subsidence scenario of the mine area due to extraction of 30 m coal seam. (**a**) Subsided area (2 m boundary line) and (**b**) vulnerable areas considering tilt angle (.3%, yellow color) and extensile strain (.2%, magenta color). (Google^22^ image, Abobe Illustrator 10, https://www.adobe.com/products/illustrator.html).

The future subsidence was predicted by considering a 30 m thick coal extraction of the thickest (36 m) coal seam without backfilling, half-strike length, and backfilling (Fig. 11). The maximum subsidence of 15.7–17.5 m in the NUP and 8.7–10.5 m in the SLP is predicted in the mining area without backfilling. The effects of the faults and dykes were not included because the effect was expected to be negligible (Fig. 9). For proper/sustainable mine management, the mining authority might need to count on this subsidence issue. The future vulnerable areas plot (Fig. 12), considering the 0.3% tilt angle and 0.2% extensile strain on the mine area to demarcate the potential danger area, might extend up to the coal mine office area and some villages (Fig. 13) considering LTCC without backfilling.

Shorter panels (half-strike length) and backfilling by fly-ash slurry (Fig. 11) are considered a subsidence-controlling approach to reduce the vulnerability from the total extraction of the 30 m coal seam. It could reduce the areal extent and magnitude of subsidence with reduced potential damage zone on the surface. The half-strike length approach shows lower subsidence and affected areas than the half-strike approach. Moreover, the production becomes half for the half-strike approach. Backfilling might be a better option (lowest subsidence with higher production) in the geo-environmental condition. Fly ash from nearby coal power plants can be used for backfilling, reducing the amount of fly ash as waste. Moreover, there is a potential to mix CO_2_ from the power plants^35–37^ for a more sustainable solution in the future.

## Concluding remarks

The maximum observed subsidence having a noticeable areal extent due to Northern Upper Panels (NUP) and Southern Lower Panels (SLP) at the Barapukuria longwall coal mine is 5.8 m and 4.2 m, respectively, after the extraction of a 10 m thick coal seam (Fig. 6). The mining-induced subsidence was simulated by the Displacement Discontinuity Method (DDM). The numerical model considered the effects of the ground surface, mining panels, faults, and the dyke. The predicted and the observed subsidence due to the mining of NUP and SLP were compared to varying Young's modulus, and the 0.10 GPa Young's modulus was found to be the best match (Fig. 8, 0.1 GPa). The effects of the faults and the dyke in the calculation were negligible (Fig. 10b). Future subsidence was predicted by considering 30 m extraction of the thick coal seam as 15.7–17.5 m in NUP and 8.7–10.5 m in SLP (Fig. 11). The potential vulnerable future zone due to the extraction might go up to the mining office area and some villages (Fig. 13). For the total extraction of the 30 m coal seam, the mining authority might need to count on this subsidence issue and adopt backfilling or other mining methods to avoid the damage of the surface structures and the land area. The research method and outcomes of the research will be helpful for proper mine management of the other coal basins considering the geo-environment conditions.

## Data Availability

The authors confirm that the data supporting the findings of this study are available within the article. The raw data that support the findings of this study are available from the corresponding author, upon reasonable request.
